# Supplementary material for: Effect of accentuated eccentric loading countermovement jumps and drop jump training with ladder training versus ladder training alone on sprint performance and change of direction ability in futsal players: A randomized controlled trial protocol
Source: PLoS One. 2026 Mar 19;21(3):e0343869. doi: 10.1371/journal.pone.0343869 (PMC13001957; doi:10.1371/journal.pone.0343869)
Supplement: S1 File — https://figshare.com/s/0d04625263b02b203d2a. (DOCX) [file pone.0343869.s001.docx]

Darpan Chaudhari

Ravi Nair Physiotherapy College, Sawangi, Wardha Sports Department

Datta Meghe Institute of Higher Education and Research Wardha, Maharashtra, India

Email: [dncjal@gmail.com](mailto:dncjal@gmail.com) Phone: +917020758710

Date: 28/01/2026

Editorial Office PLOS ONE

Dear Editors,

I am pleased to resubmit our manuscript titled *“Effect of Accentuated Eccentric Loading Countermovement Jumps and Drop Jump Training with Ladder Training versus Ladder Training Alone on Sprint Performance and Change of Direction Ability in Futsal Players: A Randomized Controlled Trial Protocol”* for consideration as a Study Protocol in PLOS ONE.

This protocol outlines a randomized controlled trial comparing two evidence-based training interventions, accentuated eccentric loading countermovement jumps and drop jumps, combined with ladder training versus ladder training alone, on sprint performance and change-of-direction ability in futsal players. At the time of this resubmission, participant recruitment has not yet begun. Recruitment is scheduled to start on 01/07/2025 and continue until 30/09/2025, with the study concluding by 30/06/2026. These timelines, along with other trial details, have been updated in the Methods section of the manuscript according to PLOS ONE guidelines for Study Protocols.

# Competing Interests:

I have read the journal’s policy, and the authors of this manuscript declare that no authors have competing interests. This does not alter our adherence to PLOS ONE policies on sharing data and materials.

# Data Availability:

Data will be securely stored under the supervision of the chief investigator, in accordance with institutional ethics approval. Upon publication of the final manuscript, the fully de-identified individual participant dataset and accompanying statistical analysis scripts will be deposited in the Figshare repository and made publicly available. A permanent digital object identifier (DOI) will be assigned at the time of publication to ensure accessibility and traceability.

Sincerely,

# Darpan Chaudhari (MPT 1st year)

On behalf of all co-authors

Ravi Nair Physiotherapy College, Sawangi, Wardha
