# Supplementary material for: Effect of accentuated eccentric loading countermovement jumps and drop jump training with ladder training versus ladder training alone on sprint performance and change of direction ability in futsal players: A randomized controlled trial protocol
Source: PLoS One. 2026 Mar 19;21(3):e0343869. doi: 10.1371/journal.pone.0343869 (PMC13001957; doi:10.1371/journal.pone.0343869)
Supplement: S3 File — https://figshare.com/s/1cf7a70e82833904420d. (PDF) [file pone.0343869.s003.pdf]

DATTA MEGHE INSTITUTE OF HIGHER EDUCATION & RESEARCH
[DEEMED to be UNIVERSITY]
Formerly known as Datta Meghe Institute of Medical Sciences (Deemed To Be University)
Re-accredited with NAAC Grade A++

INSTITUTIONAL ETHICS COMMITTEE

DCGI Re-regd. No. ECR/440/Inst/MH/2013/RR-2019
DHR Regd. No. EC/NEW/INST/2023/MH/0340
Sawangi (Meghe), Wardha-442 107, Maharashtra, India
Ph. No. : 07152 - 287701, 287702, 287703, 287704, 287705, 287706, 254501
Email :- iec.dmiher@gmail.com, Website : www.dmiher.edu.in

Ref.No. DMIHER(DU)/IEC/2025/611

Date: 08/02/2025

CERTIFICATE OF APPROVAL

The Institutional Ethics Committee in its meeting held on **30th January 2025** has approved the following research proposed to be carried out at Ravi Nair Physiotherapy College, Datta Meghe Institute of Higher Education and Research (DU), Sawangi (Meghe), Wardha.

This approval has been granted on the assumption that the proposed research work will be carried out in accordance with the ethical guidelines prescribed by Central Ethics Committee on Human Research (C.E.C.H.R.)

The details of the proposed research project for dissertation work of postgraduate program in Master of Physiotherapy (MPT) degree course discussed and approved by Institutional Ethics Committee, DMIHER(DU) are as under:-

**Name of the student** Research worker	**Name of Guide** Guidance/Supervision	**Name of Instt.** Name of Dept.	**Title of the proposed research** (PG thesis topic)	**Category** (Specialization) in subject	Approval Status
**Dr. Darpan N. Chaudhari**	Dr. Swapnil U. Ramteke	**RNPC** Dept. of Sports Physiotherapy	Effect of Accentuated Eccentric Loading Countermovement Jumps and Drop Jump Training with Ladder Training vs Ladder Training alone on Sprint Performance and Change of Direction Ability on Futsal Players: A Randomized Controlled Trial.	**PG-Thesis** for [Master of Physiotherapy] **MPT** in **Sports Physiotherapy**	**Approved**

(Dr. Swanand Pathak)
Member Secretary
Institutional Ethics Committee
DMIHER (DU)

D. M. I. H. E. R. (D.U.) — APPROVED — INSTITUTIONAL ETHICS COMMITTEE

Copy to :-

1. Dr. Darpan N. Chaudhari, MPT student
2. Dr. Swapnil U. Ramteke, Professor and Guide, Dept. of Sports Physiotherapy, RNPC
3. Principal, Ravi Nair Physiotherapy College
