# Supplementary material for: Effect of accentuated eccentric loading countermovement jumps and drop jump training with ladder training versus ladder training alone on sprint performance and change of direction ability in futsal players: A randomized controlled trial protocol
Source: PLoS One. 2026 Mar 19;21(3):e0343869. doi: 10.1371/journal.pone.0343869 (PMC13001957; doi:10.1371/journal.pone.0343869)
Supplement: S4 File — https://figshare.com/s/165de7842907f9361f86. (PDF) [file pone.0343869.s004.pdf]

DATTA MEGHE INSTITUTE OF HIGHER EDUCATION AND RESEARCH

[Deemed to be University]

Salod (Hirapur), Wardha, Maharashtra, India.

DEPARTMENT OF SPORTS PHYSIOTHERAPY

RAVI NAIR PHYSIOTHERAPY COLLEGE

SALOD (HIRAPUR), WARDHA

SYNOPSIS TITLE

EFFECT OF ACCENTUATED ECCENTRIC LOADING COUNTERMOVEMENT JUMPS AND DROP JUMP TRAINING WITH LADDER TRAINING VS LADDER TRAINING ALONE ON SPRINT PERFORMANCE AND CHANGE OF DIRECTION ABILITY IN FUTSAL PLAYERS: A RANDOMIZED CONTROLLED TRIAL

INVESTIGATOR

Dr. Darpan Chaudhari (PT)

First Year MPT Student, Department of Sports Physiotherapy,

Ravi Nair Physiotherapy College, Datta Meghe Institute of Higher Education and Research,(DU) Salod (Hirapur), Wardha-442004

GUIDED BY

Dr. Swapnil U. Ramteke (PT)

Professor, Department of Sports Physiotherapy,

Ravi Nair Physiotherapy College, Datta Meghe Institute of Higher Education and Research, (DU) Salod (Hirapur), Wardha-442004

Datta Meghe Institute of Higher Education and Research(Deemed to be University)

INSTITUTIONAL ETHICS COMMITTEE

Proforma for PG Thesis- MD/MS/MDS/M.Sc./Ph.D./M.Phil. /Fellowship Diploma & All research proposals-projects/synopsis

Name of the Institute: Ravi Nair Physiotherapy College

Department of Sports Physiotherapy

Name of the Principal Investigator	Dr. Darpan N. Chaudhari
Name of the Co-investigator/Guide	Dr. Swapnil U. Ramteke (PT)
Course of Study and Subject	MPT (Sports Physiotherapy)
Date of Admission	
Topic / Title of the Project	Effect of accentuated eccentric loading countermovement jumps and drop jump training with ladder training vs ladder training alone on sprint performance and change of direction ability in futsal players: a randomized controlled trial
SYNOPSIS (as per the guidelines)	Attached
Signature of the Candidate	
Mobile No., E-mail	Mobile No.:7020758710<br>E-mail ID: dncjal@gmail.com
Name and Designation of the Guide Mobile No. & E-mail	Dr. Swapnil U. Ramteke (PT)<br>Professor, Department of Sports Physiotherapy, Ravi Nair Physiotherapy College, Datta Meghe Institute of Higher Education and Research, Salod (Hirapur), Wardha-442004.<br>Mobile: 7619941787<br>E-mail: swapnil.ramteke@dmiher.edu.in
Signature of Guide	
Technical Soundness (rationale, literature review, objective, methodology, references)	
Feasibility (If any other Dept. & Institute is involved name & signature of the head)	Technical:<br>Infrastructural:<br>Financial:
Remarks & Signature of Chairman Departmental Research Committee	

Datta Meghe Institute of Higher Education and Research

(Deemed to be University)

INSTITUTIONAL ETHICS COMMITTEE

Clearance from Scientific Scrutiny Committee/ Institutional Research Committee

Name of the Principal Investigator	Dr. Darpan Chaudhari
Department	Sports Physiotherapy
Name of the Institute	Ravi Nair Physiotherapy College
Course of Study and Subject	MPT (Sports Physiotherapy)
Date of Admission	
Title of Project	Effect of accentuated eccentric loading countermovement jumps and drop jump training with ladder training vs ladder training alone on sprint performance and change of direction ability in futsal players: a randomized controlled trial
SYNOPSIS (as per the guidelines)	Attached
Signature of the Candidate	
Mobile No., E-mail	Mobile No.:7020758710<br>E-mail ID: dncjal@gmail.com
Name and Designation of the Guide	Dr. Swapnil U. Ramteke (PT)<br>Professor, Department of Sports Physiotherapy, Ravi Nair Physiotherapy College, Datta Meghe Institute of Higher Education and Research, Salod (Hirapur), Wardha-442004.<br>Mobile No.: 7619941787<br>E-mail ID: swapnil.ramteke@dmiher.edu.in
Signature of Guide	
Technical Soundness (rationale, literature review, objective, methodology, references)	
Feasibility	Technical:<br>Infrastructural:<br>Financial:
Remarks & Signature of Chairman Scientific Scrutiny Committee	

CENTRAL RESEARCH LABORATORY

Proforma of Feasibility Criteria for Investigations

Name of Principle Investigator (PI): Dr. Darpan Chaudhari

Name of Guide: Dr. Swapnil U. Ramteke (PT)

Department: Department of Sports Physiotherapy

Title of Project: Effect of accentuated eccentric loading countermovement jumps and drop jump training with ladder training vs ladder training alone on sprint performance and change of direction ability in futsal players: a randomized controlled trial

Sr. No.	Investigation required	Quantity	Laboratory in which the Investigation will be carried out	Signature of Laboratory In-charge
1				
2				
3				
4				
5				

Signature of PI

Signature of Guide

Signature of CRL Representative

Datta Meghe Institute of Higher Education & Research
(Deemed to be University)

INSTITUTIONAL ETHICS COMMITTEE

Submission of Research synopsis/proposal

Ethics Review Checklist

INDEX

[√ please select-Attached]

- [√] Synopsis along with the forms of the IEC
- [√] Provisional Registration Form for Ph.D. (Doctor of Philosophy) in the Faculty of Medicine/ Dentistry/ Ayurveda/ Nursing/ Interdisciplinary Sciences/ Physiotherapy.
- [√] Proforma of IEC (duly signed by Head of departmental research committee & Scientific Scrutiny Committee Chairman)
- [√] Sample size justification
- [√] Case history proforma (if applicable)
- [√] Key articles
- [√] Informed consent forms (English & Hindi/Marathi)
- [√] Budget disclosure form

Index

Sr. No.	Topic	Page No.
1.	Introduction	6
2.	Aim and Objective	14
3.	Review Of Literature	8
4.	Methodology	14
5.	Research Question	14
6.	Hypothesis	13
7.	Aim Of The Study	14
8.	References	24
9.	Annexure I – Data Assessment Sheet	26
10.	Annexure II – Data Collection Sheet	27
11.	Annexure III- Consent Form(A,B,C)	28

Effect of accentuated eccentric loading countermovement jumps and drop jump training with ladder training vs ladder training alone on sprint performance and change of direction ability in futsal players: a randomized controlled trial

I. Introduction:

Futsal is a fast-paced form of small-sided football officially recognized by FIFA. Sport that requires rapid sprints and frequent changes of direction (COD), with 5 players in a team with unlimited substitutions allowed during competitions. As a result, the game's physical demands may be extremely high. Researchers' interest in futsal has increased in recent years.(1). During in-play time, players cover over 130 m per minute, totalling over 3300 m every match, with more than 7.5% covered at high intensity (15.5 to 18.3 km·h). Compared to football, it is observed that futsal players change direction more frequently throughout a game while dribbling a ball across a reduced court size. For these reasons, COD may be a particularly relevant physical fitness criterion in futsal.(2)

Experts in Research and practitioners have incorporated eccentric-based training to properly load the eccentric motion by removing the restriction of concentric force output. The magnitude of the mechanical stimulation usually determines the skeletal muscle response, and it has been demonstrated that eccentric-only training increases response, particularly in terms of strength and size changes. Eccentric-only training has also been found to recruit motor units with high thresholds preferentially. However, due to insufficient stretch-shortening cycle (SSC) involvement and a lack of task-specificity, eccentric-only training may not be as transferable to athletics. (3) Recently, increased emphasis has been placed on sports performance and the application of accentuated eccentric loading (AEL). AEL involves using eccentric and concentric contractions to administer an eccentric load greater than the necessary concentric load while attempting to preserve natural movement patterns.(4) For instance, a coach may load a back squat with a specific weight for the eccentric section and then manually remove the weight before beginning the concentric action. This strategy is thought to improve adaptation by increasing eccentric loading, which results in more eccentric and concentric force output. Evidence shows that this training strategy causes shifts to quicker myosin heavy chain (MHC) isoforms and more favourable modifications in bispecific muscle.(5) Increases in force and power production have frequently accompanied these modifications. Furthermore, past research reveals beneficial changes in jumping and throwing movements, indicating AEL may transfer well to sport demands and performance when applied to plyometric training exercises.(6)

Furthermore, chronic AEL CMJ training has regularly exceeded traditional CMJ training in developing lower body power (+20% vs. +1%) and vertical jump height (+11% vs. -2% over time).(7). To our knowledge, no research has established if AEL applied to a CMJ can increase other independent measures of physical performance, such as lower body strength, sprinting, and cognitive performance. (8). The drop jump (DJ) involves stepping off a raised platform and leaping right off the ground. The countermovement leap (CMJ), on the other hand, starts from a standing position and accelerates explosively upward after a downward movement. According to earlier studies, the DJ can significantly produce higher force (+2.3 to 31.7%), power production (+0.8%), and jump height (+6.4 to 13.2%) than the CMJ (9). With these advantages, DJ training has effectively increased sprint performance, lower body

strength, vertical jump height, lower body power, and change of direction (COD) ability. This enhancement is attributed to the DJ increased eccentric loading phase, which enhances motor unit recruitment and activation and potential kinetic energy storage and usage.(10).

The agility ladder is a reasonably priced and simple training aid that enables coaches and athletes to be creative in adjusting task restrictions during drills and cultivating the movement coordination patterns common in team sports. Ladder workouts are designed to enhance and improve athletic performance and footwork. It improves foot speed, mobility, proprioception, agility, power, strength, balance and coordination, and response time.
(11). Because ladder drills encourage the body and mind to synchronise different foot movements, training sessions are more engaging when conducted rhythmically. Depending on the training objective, a ladder has two straps and rungs spaced 15 to 18 inches apart. Like an agility ladder, a ladder can be constructed at home with PVC pipe and rope, then taped to the floor. Ladder training helps players become more coordinated and catch, hit, block, and tackle more easily. Leaps are performed swiftly, in various directions, and without any obstacles on the agility ladder. Running through a ladder, skips, shuffles, jumps/hops, and linear and lateral movements are the four fundamental skills of ladder training.(12).

II. Need of the study:

1. Prevalence and impact:

While AEL and DJ training have been shown to improve vertical jump performance and lower body strength, there is limited evidence regarding their impact on sprint performance and change of direction (cod) ability. These are critical components of athletic performance, particularly in sports requiring explosive power, agility, and quick direction changes.(5)

2. Available solution
given that both AEL and DJ training have shown comparable effects on vertical jump and strength, a more systematic investigation is needed to assess which training method is more effective or how both could be optimally combined with other conditioning exercises to maximize athletic performance. In real-world sports settings.(6)

3. Limitations: the study aims to address a critical need to understand the combined effect of accentuated eccentric loading (AEL) CMJ, drop jump training, and ladder training on sprint performance and change of direction (cod) ability in futsal players. This research is essential for optimizing training protocols, enhancing athletic performance, and improving competitiveness in futsal, a sport requiring rapid, multidirectional movements and explosive power. By investigating these complementary methods, the study will provide valuable insights for developing more effective performance enhancement and injury prevention strategies for futsal players.(7)

III. Review of literature:

Title,author & year	Focus of study	Conclusion	Remarks
**Title:** effects of different strength and velocity training programs on physical performance in youth futsal players:<br>**Author:** oscar villanueva-guerrero,et.al(2024)	- the aim of this study was to determine the effects of different strength and velocity training programs on lower body physical performance in youth futsal players	It is observed that the strength-based training protocol has a significantly greater effect on cods compared to the velocity training program. This relationship could be linked to the importance of the eccentric phase during the landing of the cod. Therefore, the incorporation of these training protocols in the physical preparation of youth futsal players is recommended, with the aim of optimising their performance and preventing future injuries.	The results suggest that both strength and velocity training programs are effective for enhancing physical performance in youth futsal players. It showed greater improvements in change of direction capabilities, which are critical for success in futsal due to the sport's reliance on rapid directional changes and explosive movements.

**Title:** accentuated eccentric loading in countermovement jumps vs. Drop jumps:effects on jump performance and strength in a randomized controlled trial:<br>**Author:** *zhengqiu gu,et.al(2025*	This study examined the effects of accentuated eccentric loading countermovement jump (ael cmj) training on jump performance, lower body strength, sprint performance, and change of direction ability, compared to drop jump (dj) training. This study used a randomized controlled trial (rct)	Ael cmj training and dj training program effectively improved vertical jump performance and lower body strength in physically active individuals, with both training methods producing similar effects. However, neither training method significantly enhanced acceleration or change of direction (cod) performance, and no notable gains were observed beyond the first four weeks. Practitioners and coaches seeking to enhance vertical jump performance and lower body strength in athletes can incorporate either ael or dj training into their plyometric training programs, as both approaches provide comparable benefits.	- the study's findings suggest that both ael cmj and dj training are effective for enhancing vertical jump performance and lower body strength in physical education students. However, the lack of improvement in sprinting and cod abilities indicates that these training modalities may not be comprehensive enough to enhance all aspects of athletic performance.
**Title:** the science of ladder drills and plyometrics: enhancing neuromuscular efficiency in athlete<br>**Author**: pramod ravi1, divya. K (2024).	Aim of this study are to determine whether there is any interaction between agility and athletic performance skills and plyometric training, ladder drill, and agility training; how different these effects are from one another; and how different the effects	The post-test indicates a significant value of $p < 0.05$, indicating that the plyometric training technique, ladder drill, affects athletic performance skills ($p > 0.05$). Because the significance value indicates p of $0.006 < 0.05$, there is a significant difference between the effects of low and high agility on athletic performance skills ($p < 0.05$). There is a significant ($p > 0.05$) interaction between agility (high and low), ladder drill training	The findings indicate that following training, there is a relationship between agility and athletic performance abilities. According to the study, there is a connection between agility and athletic performance skills, and agility has a major impact on

	of low and high agility are from one another.	techniques, and plyometric training methods of athletic performance skills ($p < 0.05$).	athletic performance. Training techniques such as plyometric training and ladder drills are also related to agility. Applying the ladder drill and plyometric training techniques affects athletes' performance abilities. It has been demonstrated that doing plyometric and ladder drills may enhance one's athlete's performance ability.
**Title:** the effects of ladder training on sprint and change of direction performance **Author:** trevor p. Short,et.al (2022)	The purpose of this study was to examine the effects of ladder training (lt) on sprint (20-m) and change of direction (cod) (l-drill) kinematics.	Fourteen basketball players were matched on baseline performance rankings and randomly assigned to a lt or conventional training (ct) group. The lt group performed all the ct exercises with the addition of 25-35 minutes of lt performed 3 times per week, for 4 weeks. Within-group analyses showed significant improvements in 20-m sprint performance from pretest to post test in lt and ct. No statistically significant difference was found in cod performance. Significant	Lt resulted in substantially greater enhancements in stride frequency (es = and ground contact time in the 20-m sprint and cod performance respectively. Lt may be more effective than ct in improving performance and kinematics. Lt should be

		enhancements were found in stride frequency from pretest to post-test for 20-m sprint and cod. Lt resulted in substantially better results (between-group changes) in both the 20-m sprint and cod test furthermore,	implemented as a warmup exercise.
**Title:** the isoinertial training method owes its efficacy to an accommodated resistance and optimal individualized eccentric overload. **Author:** giovanni fiorilli,et.al(2020)	The aim of this study was to assess the effects of a 6-week isoinertial eccentric-overload training program - using a flywheel inertial device during the execution of specific soccer exercises - on explosive and reactive strength, sprint ability, change of direction (cod) performance and soccer shooting precision these results confirmed the positive effect of is inertial training.	The absence of knowledge of the eccentric overload applied by the isoinertial device, which is different in any exercise repetition, may stimulate the athlete's neural adaptations, improving their soccer skills and in particular their soccer shooting precision.	: highlights the significant implications of isoinertial eccentric-overload training for young soccer players. By utilizing a flywheel inertial device, this training method enhances explosive strength, reactive strength, sprint ability, change of direction performance, and shooting precision more effectively than traditional methods. The variability in eccentric overload stimulates beneficial

			neural adaptations, improving muscle coordination and resilience, which can reduce injury risks. Coaches are encouraged to integrate isoinertial training into their programs to optimize athletic performance and support injury prevention strategies, making it a valuable addition to soccer training regimens.

IV. Research gap analysis:

1. **Practical knowledge gap** (action-knowledge conflict gap)

Future research could consider combining ael and dj training with other types of ssc exercises to explore complementary methods for optimizing sprint and cod performance. (7)

2. **Evidence gap** (contradictory evidence)

The study found that while both accentuated eccentric loading (ael) and drop jump (dj) training improved jump performance and lower body strength, there were no significant improvements in sprinting or change of direction (cod) performance. This outcome contradicts previous research and theoretical expectations that suggested these training

methods should enhance sprint and cod abilities. This inconsistency shows a gap in evidence regarding the effectiveness of ael and dj training for these specific athletic qualities.

V. Research question:

Is there a significant effect of Effect of accentuated eccentric loading countermovement jumps and drop jump training with ladder training vs ladder training alone on sprint performance and change of direction ability in futsal players: a randomized controlled trial ?

**P** (population)	Futsal players
**I** (intervention)	Effect of accentual eccentric loading countermovement jumps and drop jump training with ladder training
**C** (comparison)	Ladder training without the combined intervention
**O** (outcome)	Sprint performance and change of direction ability
**T** (time)	1 year

VI. Hypothesis:

1. Null Hypothesis :

H_{01}-there will be no effect of accentual eccentric loading countermovement jumps and drop jump training with ladder training vs ladder training alone on sprint performance and change of direction ability in futsal players

H_{02}-there will be no significant difference beetween effect of accentual eccentric loading countermovement jumps and drop jump training with ladder training vs ladder training alone on sprint performance and change of direction ability in futsal players: a randomized controlled trial

Alternative Hypothesis(ha)

H_1-there will be significant effect of accentual eccentric loading countermovement jumps and drop jump training with ladder training vs ladder training alone on sprint performance and change of direction ability in futsal players: a randomized controlled trial

H2-there will be significant difference between effect of accentual eccentric loading countermovement jumps and drop jump training with ladder training vs ladder training alone on sprint performance and change of direction ability in futsal players: a randomized controlled trial

VII. Aim of the study:

The aim of this study is to find out of effect of accentuated eccentric loading countermovement jumps, drop jump training vs ladder training alone on sprint performance and change of direction ability in futsal players.

VIII. Objectives of the study:

- To evaluate the effect of ladder training on sprint performance and change of direction ability in futsal players.
- To evaluate the effect of accentuated eccentric loading cmj, drop jump training with ladder training on sprint performance and change of direction ability in futsal players.
- To compare the effect of accentuated eccentric loading countermovement jumps and drop jump training with ladder training vs ladder training alone on sprint performance and change of direction ability in futsal players.

IX.Methodology:

Study type: Experimental study

Study design: Randomised controlled trial (RCT)

Target population: Futsal players

Sampling technique: Simple random sampling

Duration of study: 1 year

Allocation ratio: 1:1

Blinding: Single blinded (participants will be blinded)

Study setting : futsal turfs across dmiher , Sawangi Meghe Wardha

Study population: recreational and competitive futsal players

Sample size: the sample size for this study was determined using g*power (version 3.1.9.4, heinrich heine universität düsseldorf, düsseldorf, germany), a statistical software that facilitates power analysis and sample size estimation based on various statistical tests.

formula using mean difference

$n1 = n2 = 2 \times [(z\alpha + z\beta)^2 \times \sigma^2] / (\delta^2)$

Where:

- $Z\alpha = 1.96$ (considering ci at 95%)
- $Z\beta = 1.64$ (power at 95%)

Primary variable: 30 m sprint

- Mean ± sd (pre) result on 30 m sprint in tbi readings = 4.36 ± 0.12
- Mean ± sd (post) result on 30 m sprint in tbi readings = 4.29 ± 0.13
- Mean difference (δ) = 0.064
- Pooled standard deviation = 0.125

Minimum sample size required

$N1 = n2 = 2 \times [(1.96 + 1.64)^2 \times (o.125)^2] / (0.125)^2 = 28$ per group.

Considering a 10% dropout rate:

- Dropout = 10%
- **Total sample size required =** 62.

X. Materials required:

1. Data collection sheet
2. Data assessment sheet
3. Informed consent
4. Wooden jump box
5. Dumbbells (cockatoo)
6. A stopwatch or timing gates
7. Measuring tape

8.cones and tape to mark the start and finish lines

9.agility ladder

XI. Tools and instrument

1. **Measuring tape**: used to mark accurate distances for sprint and agility tests.
2. **Cones/markers**: define start/finish lines and layouts for agility tests.
3. **Stopwatch**: manual timing alternative for sprints and agility drills.
4. **Dumbbells for accentual eccentric loading**: used to provide controlled resistance during the lowering (eccentric) phase of countermovement jumps, emphasizing gradual muscle lengthening under load.
5. **Agility ladder**: used for ladder drills to enhance agility and footwork.
6. **Video cameras**: record movements for biomechanical analysis of speed and cod.
7. **T-test setup**: standardized cone layout for lateral and forward cod assessment.
8. **Data sheets**: for recording, organizing, and analysing performance data.

XII. Variables:

dependent variables:

a. 30m sprint time: this variable measured sprint performance, allowing for the evaluation of speed improvements resulting from the training.

b. T-test for agility: this test assessed change of direction ability, which is crucial for many sports and physical activities

Independent variable:

a. Accentuated eccentric loading cmj, drop jump training adjunct to ladder training
b. Ladder training

XIII. Eligibility criteria:

- **Inclusion criteria:**
  (1) Age between 18-30 years
  (2) BMI-18.5–24.9
  (3) Regular participation in futsal activities since 1 year.
  (4) Recreational and competitive futsal players participating in regional, national, or professional leagues
  (5) Willingness to participate in a structured 6-week intervention program focusing on accentuated eccentric loading Countermovement jumps, drop jump training, and ladder drills.
- **Exclusion criteria:**
  (1) presently having pain due to trauma associated to lower limb
  (2) laxity of articular ligaments or meniscus injury around knee
  (3) recent history of dislocation of patella or recent fracture
  (4) chondromalacia patella
  (5) existing progressive neurological conditions
  (6) plantar fasciitis

XIV. Procedure:

Participants will be screened based on the inclusion and exclusion criteria. Individuals meeting the inclusion criteria will be recruited for the study after being informed about the purpose and nature of the study. Written informed consent will be obtained from all participants. Participants will then be randomly divided into two groups: group a and group b, with equal participants in each group.

Before the intervention, both groups will undergo baseline assessments for sprint performance and change of direction (cod) ability. Once baseline testing is completed, participants will receive their respective interventions as described below:

- Group A: Accentuated eccentric loading (AEL) countermovement jumps, drop jump training with ladder training.
- Group B: Ladder training only.

Group A will receive accentuated eccentric loading (AEL) countermovement jumps and drop jump training with ladder training and Group B will receive Ladder training.

XV. CONSORT FLOW CHART

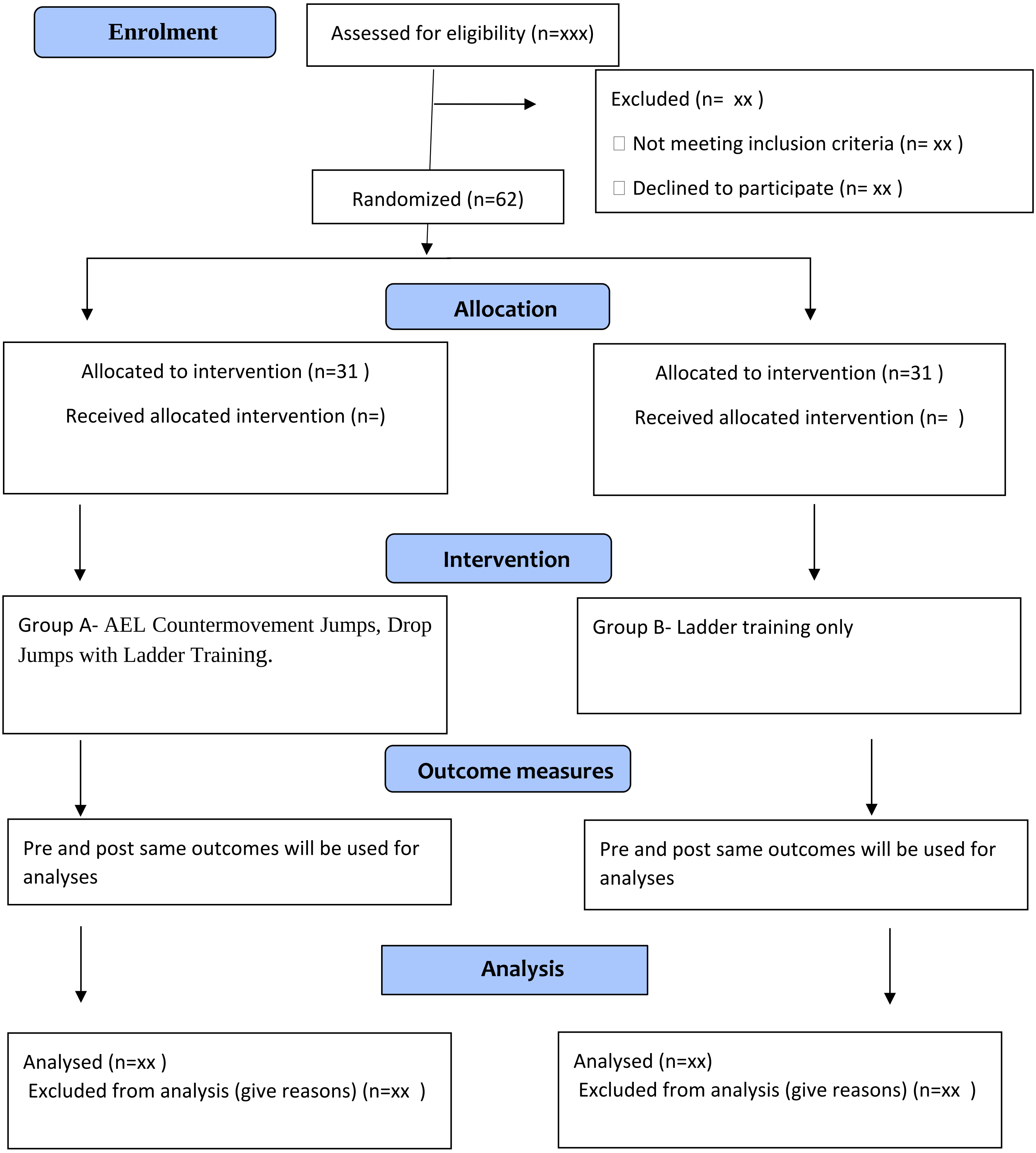

Participant timeline:

Once ethical approval is obtained from the university ethical committee, the subjects will be recruited from the RNPC Department of Sports Physiotherapy. After receiving consent from the subjects, they will be assessed and then will be divided into 2 groups.

Group A:

Accentuated eccentric loading Countermovement jump, drop jumps with Ladder training

Exercise category	Procedure:	Reps/sets and duration	Progression
Accentuated eccentric loading (ael) countermovement jumps	- use dumbbells or a weighted harness for eccentric loading.<br>- perform countermovement to approximately 90° knee flexion.<br>- drop the load before the concentric phase for maximal jump.	**Weeks 1-3:** 4 sets of 8 repetitions<br>**weeks 3-6:** 4 sets of 10 repetitions<br>(3 sessions per week)	Start with 10-20% of body mass as additional eccentric load, progressively increasing based on tolerance. Rest: 2 minutes between sets.
Drop jump training	**Procedure:**<br>- step off the box without jumping.<br>- land softly on the balls of the feet and immediately perform a vertical jump.	**Weeks 1-3:** 4 sets of 8 repetitions<br>**weeks 3-6:** 4 sets of 10 repetitions<br>(3 sessions per week)	Drop height: begin with 30 cm, progressing to 50 cm as tolerated. Rest: 2 minutes between sets.
Ladder training	**Drills:**<br>- **weeks 1-2:** straight run, hopscotch, single-foot hops zigzag pattern.<br>- **weeks 3-4:** two-foot run, backward hopscotch, single-foot lateral in/out hops.<br>- **weeks 5-6:** bunny hops, hopscotch variation, two-foot hops zigzag pattern	6 repetitions of each drill x 2 sets per session.<br>(3 sessions per week)	Rest: 30 seconds between repetitions, 60 seconds between sets. Focus on quick ground contact, coordination, and multidirectional movement.

Group B: control group

**Exercise category**	**Procedure:**	**Reps/sets and duration**	**Progression**
**Ladder training**	**Drills:**<br>- **Weeks 1-2:** straight run, hopscotch, single-foot hops zigzag pattern.<br>- **Weeks 3-4:** two-foot run, backward hopscotch, single-foot lateral in/out hops.<br>- **Weeks 5-6:** bunny hops, hopscotch variation, two-foot hops zigzag pattern.	6 repetitions of each drill × 2 sets per session.<br>(3 sessions per week)	**Rest:** 30 seconds between repetitions, 60 seconds between sets.<br>Focus on quick ground contact, coordination, and multidirectional movement.

XVI. Outcome measures:

Primary outcome measure

- **30 M Sprint Test-**

this study investigates the validity and reliability of various timing systems used in a 30-meter sprint test, focusing on their precision in performance evaluation. The systems analyzed include manual stopwatch timing.

Key findings:

- Manual timing: icc ranged from 0.60 to 0.75, showing moderate reliability but significant errors due to human reaction times.

The study concludes that fully automated systems are the most accurate and reliable for sprint performance measurement, ensuring consistency and reducing errors. These findings are crucial for sports performance testing, enabling coaches and researchers to make informed decisions about timing methods.

- **Agility T-Test-**
  The agility t-test assesses an athlete's ability to accelerate, decelerate, and change direction quickly while maintaining control. It consists of a series of movements: sprinting forward, shuffling laterally, and backpedalling, typically completed in under 9.5 seconds for men and 10.5 seconds for women to be considered excellent. The test evaluates leg speed, power, and agility, making it a reliable measure for athletic performance across various sports. While it primarily measures change of direction, it also reflects overall athleticism and movement efficiency.
  The test-retest reliability of the agility t-test is generally considered high. A study indicated an intraclass correlation coefficient (icc) of 0.84, suggesting that the test yields consistent results across multiple trial

XVII. Data collection, management, and analysis

1. Data collection methods

Pre-intervention data will be collected upon enrolment; additional post-intervention data will be collected after 6 weeks of the intervention.

		Study period							
	Enrolment	Allocation	Post-allocation						Close-out
Timepoint**	$-t_1$	0	T_1	T_2	T_3	T_4	T_5	T_6	T_x
**Enrolment:**									
**Eligibility screen**	X								
**Informed consent**	X								
[list other procedures]	X								
**Allocation**		X							
**Interventions:**									
[intervention a]									
[intervention b]									
[list other study groups]									
**Assessments:**									
[list baseline variables]	X								
[list outcome variables]			X		X		X		X
[list other data variables]			X		X		X		X

2. Data management

Collected information will be summarized using basic descriptive statistics, including frequency percentages for qualitative data and mean with standard deviation for quantitative data.

3. Statistical method

The complete analysis data set will include all the participants in the study with no missing values for all the parameters in the data set. The participants will be the subjects fulfilling the inclusion and exclusion criteria.

Outcome variables: Primary outcomes

1. 30 Meter Sprint Test
2. Agility T-Test

The gathered information will be condensed to produce baseline characteristics based on demographic factors. Continuous variables will be represented by mean and standard deviation; categorical variables will be represented by frequency and percentage. The continuous variables will be tabulated with the mean, standard deviation, standard error, minimum, maximum, and confidence interval (CI) at 95% for parametric data after being compared to the outcome variables with a 5% significance level ($P \leq 0.05$), data from all continuous outcome variables will be tested using a normality test called Kolmogorov-Smirnov test. Non-parametric tests will be used to assess significance if the null hypothesis of normality is rejected, implying that the data is not normal. The significant difference will be at a 5% level ($P \leq 0.05$) using the t-test.

For comparison groups:

1. Accentual Eccentric Loading Countermovement Jumps and Drop Jump Training with Ladder Training
2. Ladder Training Alone

the Mann-Whitney U test will be used to determine significance and The mean, median, upper quartile and lower quartile will be used to characterize the variables in non-normal data. Frequent (N) and percentage (%) will summarize categorical variables. Chi-square analysis will be utilized to examine efficacy over categorical variables.

Methods monitoring

Data monitoring: The data will be monitored by the data monitoring committee of Ravi Nair Physiotherapy College.

Harms:
Any episode of the adverse events shall be reported to the ethical committee and the clinician in charge for assessing and managing the solicited and spontaneous adverse events and other unintended effects of trial interventions or trial conduct.

XVIII. Scope and implication of the study

Scope of study

limited studies have investigated effect of accentuated eccentric loading countermovement jumps and drop jump training with ladder training vs ladder training alone on sprint performance and change of direction ability in futsal player of which leads to difficulty in the definitive conclusions about the interventions in futsal players. This made it necessary to study, with greater standardization, type of intervention, treatment protocol, and with a larger sample size to see the improvement in their performance.

Implication of study

Effect of accentuated eccentric loading countermovement jumps and drop jump training with ladder training vs ladder training alone on sprint performance and change of direction ability in futsal players: a randomized controlled trial

Which is very much essential effect of both training protocols could suggest a new therapeutic method.

XIX. References:

1. Barbero-Alvarez JC, Soto VM, Barbero-Alvarez V, Granda-Vera J. Match analysis and heart rate of futsal players during competition. Journal of Sports Sciences. 2008 Jan;26(1):63–73

2. Sekulic D, Foretic N, Gilic B, Esco MR, Hammami R, Uljevic O, et al. Importance of Agility Performance in Professional Futsal Players; Reliability and Applicability of Newly Developed Testing Protocols. Int J Environ Res Public Health. 2019 Sep 4;16(18):3246.

3. Merrigan JJ, Tufano JJ, Falzone M, Jones MT. Effectiveness of Accentuated Eccentric Loading: Contingent on Concentric Load. International Journal of Sports Physiology and Performance. 2021 Jan 1;16(1):66–72.

4. Wagle JP, Taber CB, Cunanan AJ, Bingham GE, Carroll KM, DeWeese BH, et al. Accentuated Eccentric Loading for Training and Performance: A Review. Sports Med. 2017 Dec;47(12):2473–95.

5. Walker S, Blazevich AJ, Haff GG, Tufano JJ, Newton RU, Häkkinen K. Greater Strength Gains after Training with Accentuated Eccentric than Traditional Isoinertial Loads in Already Strength-Trained Men. Front Physiol [Internet]. 2016 Apr 27 [cited 2025 Apr 26];7. Available from: https://www.frontiersin.orghttps://www.frontiersin.org/journals/physiology/articles/10.3389/fphys.2016.00149/full

6. Merrigan J, Borth J, Taber C, Suchomel T, Jones M. Application of Accentuated Eccentric Loading to Elicit Acute and Chronic Velocity and Power Improvements: A Narrative Review. International Journal of Strength and Conditioning. 2022 Feb 3;2(1).

7. Accentuated eccentric training: effects on horizontal jump distance and muscle strength among young adults. MOJ Yoga Phys Ther [Internet]. 2018 Jun 12 [cited 2025 Apr 26];Volume 3(Issue 3). Available from: https://medcraveonline.com/MOJYPT/MOJYPT-03-00045.pdf

8. Wagle JP, Taber CB, Cunanan AJ, Bingham GE, Carroll KM, DeWeese BH, et al. Accentuated Eccentric Loading for Training and Performance: A Review. Sports Med Auckl NZ. 2017 Dec;47(12):2473–95.

9. Godwin MS, Fearnett T, Newman MA. The Potentiating Response to Accentuated Eccentric Loading in Professional Football Players. Sports. 2021 Nov 26;9(12):160.

10. Gu Z, Gao C, Zheng H, Liao K, Bishop C, Hughes J, et al. Accentuated Eccentric Loading in Countermovement Jumps Vs. Drop Jumps: Effects on Jump Performance and Strength in A Randomized Controlled Trial. J Sports Sci Med. 2025 Mar 1;24(1):20–30.

11. Gatz G. Complete Conditioning for Soccer. Human Kinetics; 2009. 210 p.

12. Prakash KVS, Sadvika PD, Chakravarthi CA. Effectiveness of Ladder Training Versus Plyometric Training Program on Agility in Kabaddi Players. International Journal of Health Sciences and Research. 2021 Nov 29;11(11):320–34.

XX. ANNEXURE I (DATA ASSESSMENT SHEET)

Name:

Age:

Gender:

Address and contact number:

History:

Medications:

Status: Patient Included ☐ Excluded ☐

Signature of Investigator

Signature of Co-Investigator

XXI. ANNEXURE II

(DATA COLLECTION SHEET)

Unique ID:

Age:

Sex: Male ☐ Female ☐

Phone No:

Date:

Outcome measures	Pre	Post
**30 m sprint (s)**		
**Agility T Test**		

Annexure – III A

Consent form – English

Study title: effect of Accentuated eccentric loading countermovement jumps and drop jump training with ladder training vs ladder training alone on sprint performance and change of direction ability in futsal players: a randomized controlled trial

Name of investigator: dr. Darpan Chaudhari (pt)

Name of guide: dr. Swapnil Ramteke (pt)

This is to certify that i………………………………………………………………. Have been given the required information concerning my participation as a volunteer in the above-mentioned study. The contents of the form have been explained to me in my own language. I confirm that i will receive a signed copy of the consent form. I have understood the nature of the study and i volunteer to participate in this research study as a subject.

Name: -…………………………………………………………….

Age/gender:

Address:

Contact no:

Date: / /20

Sign:

Place:

I undersigned Dr. Darpan Chaudhari (pt) has explained the study details and have cleared all the quires put forth by the above volunteer to the best of my ability. I confirm that all data and test results achieved will be kept strictly confidential and will be withheld from any misuse.

Date: / /20

Sign:

Place

Annexure - III B

सहमति पत्र - हिंदी

अध्ययन का शीर्षक: **टसाल खिलाड़ियों में स्प्रिंट प्रदर्शन और दिशा बदलने की क्षमता पर लहजे वाले एक्सेंट्रिक लोडिंग , ड्रॉप जंप ट्रेनिंग और सीढ़ी प्रशिक्षण के संयोजन का प्रभाव: एक यादृच्छिक नियंत्रित परीक्षण।**

का नाम: डॉ. दर्पण चौधरी (पीटी)

गाइड का नाम: डॉ. स्वप्निल रामटेके (पीटी)

प्रमाणित किया जाता है कि मैं .... उपर्युक्त अध्ययन में एक स्वयंसेवक के रूप में मेरी भागीदारी के संबंध में आवश्यक जानकारी दी गई है। फॉर्म की सामग्री मुझे मेरी अपनी भाषा में समझा दी गई है। मैं पुष्टि करता हूं कि मुझे सहमति प्रपत्र की एक हस्ताक्षरित प्रति प्राप्त होगी। मैंने अध्ययन की प्रकृति को समझ लिया है और मैं स्वेच्छा से एक विषय के रूप में इस शोध अध्ययन में भाग लेता हूं।

नाम: -..।

उम्र और लिंग:

पता:

संपर्क नंबर:

दिनांक: / /20

हस्ताक्षर:

जगह:

मेरे अधोहस्ताक्षरी डॉ. दर्पण चौधरी (पीटी) ने अध्ययन विवरण समझा दिया है और अपनी सर्वोत्तम क्षमता के अनुसार उपरोक्त स्वयंसेवक द्वारा रखी गई सभी जिज्ञासाओं का समाधान

कर दिया है। मैं पुष्टि करती हूं कि प्राप्त किए गए सभी डेटा और परीक्षण परिणामों को सख्ती से गोपनीय रखा जाएगा और किसी भी दुरुपयोग से रोका जाएगा।

दिनांक: : / /20

हस्ताक्षर:

Annexure – III C

संमती पत्र - मराठी

फुटसाल खेळाडूंच्या स्प्रिंट कामगिरी आणि दिशा बदलण्याच्या क्षमतेवर ॲक्सेंच्युअल एक्सेंट्रिक लोडिंग, ड्रॉप जंप ट्रेनिंग आणि जिन्याच्या प्रशिक्षणाचा संयुक्त प्रभाव: एक यादृच्छिक नियंत्रित चाचणी। तपासनीसाचे

नाव: **डॉ.** डॉ. दर्पण चौधरी (पीटी)

मार्गदर्शकाचे नाव: डॉ.स्वप्नील रामटेके (पीटी)

हे प्रमाणित करण्यासाठी आहे की मी .... वर नमूद केलेल्या अभ्यासात स्वयंसेवक म्हणून माझ्या सहभागासंबंधी आवश्यक माहिती दिली आहे. फॉर्ममधील मजकूर मला माझ्या भाषेत समजावून सांगितला आहे. मी पुष्टी करतो की मला संमती फॉर्मची स्वाक्षरी केलेली प्रत मिळेल. मला अभ्यासाचे स्वरूप समजले आहे आणि मी एक विषय म्हणून या संशोधन अभ्यासात सहभागी होण्यासाठी स्वयंसेवक आहे.

नाव:-...

वय/लिंग:

पत्ता:

संपर्क क्रमांक:

दिनांक : / /20

स्वाक्षरी:

ठिकाण:

मी खाली स्वाक्षरी केलेले डॉ. दर्पण चौधरी पीटी) यांनी अभ्यासाचे तपशील स्पष्ट केले आहेत आणि वरील स्वयंसेवकाने मांडलेल्या सर्व प्रश्नांना माझ्या क्षमतेनुसार पूर्ण केले आहे. मी पुष्टी करते की प्राप्त केलेला सर्व डेटा आणि चाचणी परिणाम काटेकोरपणे गोपनीय ठेवले जातील आणि कोणत्याही गैरवापरापासून रोखले जातील.

दिनांक: / /20

स्वाक्षरी:

ठिकाण:
