# Supplementary material for: Effect of accentuated eccentric loading countermovement jumps and drop jump training with ladder training versus ladder training alone on sprint performance and change of direction ability in futsal players: A randomized controlled trial protocol
Source: PLoS One. 2026 Mar 19;21(3):e0343869. doi: 10.1371/journal.pone.0343869 (PMC13001957; doi:10.1371/journal.pone.0343869)
Supplement: S5 File — https://figshare.com/s/068bc0d3674d130cb312. (PDF) [file pone.0343869.s005.pdf]

S5 File – Intervention Fidelity Checklist

**Fidelity Item**	**Session 1**	**Session 2**	**Session 3**	**Session 4**
Session Date				
Participant ID				
Warm-up Completed (Yes/No)				
Ladder Drills Correct Sets/Reps (Yes/No)				
Drop Jumps Correct Sets/Reps/Height (Yes/No)				
AEL CMJ Load Correct (%BM) (Yes/No)				
Rest Intervals Followed (Yes/No)				
Technique Observed (Yes/No/Comments)				
Total Ground Contacts Achieved				
Deviations/Notes				
Supervisor Initials				

Instructions for Use:

1. Each column after "Fidelity Item" represents a single session.
2. Fill in Session Date and Participant ID for each session.
3. Mark "Yes" or "No" for completed items, and note any deviations.
4. Add comments under Technique Observed for quality checks.
5. Record total ground contacts and supervisor initials for accountability
