# Supplementary material for: Effect of accentuated eccentric loading countermovement jumps and drop jump training with ladder training versus ladder training alone on sprint performance and change of direction ability in futsal players: A randomized controlled trial protocol
Source: PLoS One. 2026 Mar 19;21(3):e0343869. doi: 10.1371/journal.pone.0343869 (PMC13001957; doi:10.1371/journal.pone.0343869)
Supplement: Attachment — Submitted filename: Response to Reviewers.pdf [file pone.0343869.s007.pdf]

Response to Reviewers

Title: Effect of Accentuated Eccentric Loading Countermovement Jumps and Drop Jump Training with Ladder Training versus Ladder Training Alone on Sprint Performance and Change of Direction Ability in Futsal Players: A Randomized Controlled Trial Protocol

Dear Academic Editor and Reviewers,

We sincerely thank the Academic Editor and Reviewers for their thoughtful and constructive feedback on our manuscript. Your insights have helped us refine the clarity, rigor, and overall scientific quality of our study. Below, we provide a detailed, point-by-point response to each comment. Revisions made to the manuscript are highlighted in the file titled "Revised Manuscript with Track Changes."

Editorial Requests

1. Response to Reviewers Letter
Response: This document provides a comprehensive, point-by-point rebuttal addressing each comment raised by the reviewers and the Academic Editor.

2. Competing Interests Statement
Response: The statement has been updated to:
"The authors have declared that no competing interests exist. This does not alter our adherence to PLOS ONE policies on sharing data and materials."
This has also been included in the cover letter.

3. Ethics Statement Placement
Response: The ethics statement has been restricted to the Methods section and removed from other sections, in accordance with journal requirements.

4. Data Availability Statement
Response: The following statement has been included:
"All data will be made available within the manuscript and/or supporting information files."

5. Data Anonymization
Response: We confirm that all participant-level data will be fully anonymized, with personally identifiable information removed prior to sharing.

Reviewer #1 Comments

Comment 1 (Introduction – Referencing):

"There are a few issues in the introduction that could benefit from revision. First, the referencing style is inconsistent: sometimes a period is placed after the citation number, and sometimes not. For example, citation (11) appears on a separate line, disconnected from the sentence it likely refers to, which may create confusion."

Response:
We have carefully revised the entire introduction to ensure consistent referencing style. All citations now follow PLOS ONE style guidelines, and misplaced references have been corrected to appear directly after the relevant sentence.

Comment 2 (Introduction – Eccentric Training Reference):

"Second, citation (3) is used to support a statement regarding eccentric training alone; however, upon reviewing this source, it does not appear to specifically address isolated eccentric training as the main focus, even if this is mentioned briefly in the introduction. To strengthen this point, I would suggest including references that directly evaluate the effects of eccentric-only training."

Response:
We have revised the statement and included additional references that specifically investigate eccentric-only training and its effects on performance outcomes. These changes strengthen the rationale for including AEL as part of our intervention. (Revised lines: Introduction, paragraph 2, lines xx–xx in the revised manuscript.)

Comment 3 (Introduction – Study Aim & Hypotheses):

"Lastly, although this is a Registered Report Protocol, the introduction would benefit from a clearer articulation of the study's aims and hypotheses. At present, these elements are only implicitly stated and deserve more explicit formulation to frame the study rationale and the research questions more clearly."

Response:
We have rewritten the last paragraph of the introduction to explicitly state the study's primary aim and clearly present our hypothesis that AEL CMJ + DJ + ladder training will lead to greater improvements in sprint performance and change of direction ability compared to ladder training alone.

Comment 4 (Protocol – Intervention Description):

"In the 'Interventions' part, one key aspect of the protocol that would benefit from clarification is the precise content and volume of the interventions in both groups. Both groups are said to receive ladder training, yet this component is not clearly described or consistently presented in the tables outlining the intervention programs. Greater clarity is needed to confirm that both groups indeed receive an equivalent ladder training regimen."

Response:

We have revised Tables 1 and 2 to clearly describe the ladder training content for both groups, including weekly drill progressions, repetitions, sets, rest periods, and total session duration. The description now confirms that both groups receive identical ladder training.

Comment 5 (Protocol – Training Volume Balance):

"Furthermore, there appears to be a potential imbalance in training volume between Group A and Group B, as Group A performs additional exercises (AEL CMJ and drop jumps) alongside the ladder training, whereas Group B completes ladder training only. This difference may introduce a confounding factor..."

Response:
To control for training volume, we added a statement clarifying that both groups will have identical session durations (≈45 minutes), and Group B will have additional low-intensity technical drills or structured rest periods during the time Group A performs AEL CMJ and DJ. This adjustment ensures equal total training exposure and removes potential confounding due to volume imbalance. (Methods, Interventions section updated.)

Comment 6 (Discussion – Expected Outcomes):

"It would be valuable to include a short section on expected outcomes, supported by existing literature..."

Response:
We have added a concise "Expected Outcomes" section at the end of the Discussion that summarizes our rationale and expected improvements in sprint and COD performance based on prior literature.

Reviewer #2

Comment 1 (References):

"Please double check the references: sometimes it comes between dots, sometimes not."

Response:
All references have been reviewed and reformatted for consistency per PLOS ONE guidelines.

Comment 2 (Numbers):

"Please make a correction in the description of number (sometimes 3 and sometimes three)."

Response:
All numeric references in the text have been standardized (using numerals consistently, per journal style).

Comment 3 (Figure 1):

"The figure 1 is incomplete. Please provide the correct image."

Response:
Figure 1 has been revised to a complete SPIRIT figure, clearly outlining enrolment, allocation, interventions, and assessments, following SPIRIT 2013 guidelines.

Comment 4 (Discussion):

"Please elaborate more the discussion. Bring the results and discuss them."

Response:
We have expanded the discussion to include more context from previous studies, a clearer justification for our intervention, and an explicit statement of expected results and potential implications for futsal training and rehabilitation.
We appreciate the reviewers' and editor's valuable feedback. We believe that the revised manuscript now addresses all concerns and represents a significant improvement.

Kind regards,
Darpan Chaudhari (on behalf of all co-authors)
Ravi Nair Physiotherapy College
Datta Meghe Institute of Higher Education and Research
Wardha, Maharashtra, India
