# Supplementary material for: Effect of accentuated eccentric loading countermovement jumps and drop jump training with ladder training versus ladder training alone on sprint performance and change of direction ability in futsal players: A randomized controlled trial protocol
Source: PLoS One. 2026 Mar 19;21(3):e0343869. doi: 10.1371/journal.pone.0343869 (PMC13001957; doi:10.1371/journal.pone.0343869)
Supplement: Attachment — Submitted filename: Response to Reviewers 2.0 (1).pdf [file pone.0343869.s008.pdf]

Response to Reviewers

Manuscript title:
Effect of Accentuated Eccentric Loading Countermovement Jumps and Drop Jump Training with Ladder Training versus Ladder Training Alone on Sprint Performance and Change of Direction Ability in Futsal Players: A Randomized Controlled Trial Protocol

We sincerely thank the Academic Editor and Reviewers for their careful evaluation and constructive feedback. We have addressed all comments raised by Respected Reviewer #3 and Reviewer #4 in full, and we believe the manuscript has been substantially strengthened in terms of methodological clarity, statistical rigor, theoretical justification, and editorial quality. All revisions are highlighted in the revised manuscript with tracked changes.

Reviewer #3

We thank Reviewer #3 for their positive assessment of the protocol and for identifying specific points requiring clarification. Each required change has been addressed as follows.

1. Inconsistency in Statistical Analysis Plan

Comment:
There is inconsistency between the stated use of linear mixed-effects models (LMMs) and later descriptions of t-tests/Mann–Whitney tests.

Response:
We agree and have resolved this inconsistency. The primary analysis method has been clearly defined as linear mixed-effects models (LMMs) throughout the manuscript.

Revisions made:

- The Abstract, Methods, and Statistical Analysis sections now consistently state that LMMs will be used as the primary analysis.
- The model specification has been explicitly described:
  - Fixed effects: group, time, and group × time interaction
  - Random effects: participant-level random intercept
- Software and package are specified (R, *lme4* package).
- Results will be reported as adjusted group × time contrasts with 95% confidence intervals.
- Independent t-tests or Mann–Whitney U tests are now explicitly described as secondary/sensitivity analyses only, where applicable.

2. Sample Size / Power Clarification

Comment:
Power level ($Z\beta = 1.64$) and wording are unclear; effect size source and clinical relevance need justification.

Response:
We have clarified and corrected the sample size section as follows:

- Explicitly stated that the study is powered at 95% power, with justification provided due to the performance-focused nature of the outcomes and expected small effect sizes in trained athletes.
- The source study from which the mean difference (0.064 s) and pooled SD (0.125 s) were derived has been cited.
- Clinical relevance of a 0.064 s improvement in 30-m sprint performance has been justified based on futsal and court-sport performance benchmarks.
- The sample size formula has been corrected typographically with consistent notation and symbol definitions.
- Retention of the final recruited sample size (n = 62) has been justified, including allowance for ~10% attrition.

3. Missing Data, Multiple Imputation, and ITT

Comment:
The missing-data strategy lacks sufficient detail.

Response:
We have expanded the missing-data and ITT section to include:

- Variables included in the imputation model (baseline outcomes, group, time, sex, age).
- Number of imputations (m = 20).
- Software and method specified (multiple imputation by chained equations using R).
- Handling of repeated measures within participants.
- Primary inference based on pooled LMM estimates across imputations.
- Participants with no post-baseline data will remain in ITT analyses using imputed outcomes; per-protocol analyses are explicitly labeled as secondary.

4. Data Availability Statement

Comment:
The data-sharing plan is vague and inconsistent.

Response:
We have revised the Data Availability Statement to be fully compliant with PLOS ONE requirements:

- The fully de-identified individual participant dataset and statistical analysis scripts will be deposited in Figshare.
- Data will be made publicly available upon publication of the final manuscript.
- A DOI will be assigned at the time of publication.
- Ethical considerations regarding anonymization have been explicitly stated.

5. Monitoring, Interim Analysis, and Adverse Events

Comment:
Operational details of monitoring and adverse-event reporting are insufficient.

Response:
We have expanded this section to specify:

- Frequency of safety monitoring (weekly).
- Criteria for pausing or stopping participation (e.g., musculoskeletal injury related to intervention).
- Adverse event adjudication by the chief investigator and independent clinician.
- Recording procedures and reporting of harms in the final publication.
- Statement regarding institutional insurance/compensation coverage.

6. Intervention Fidelity and Monitoring

Comment:
More detail is needed on fidelity procedures and progression decisions.

Response:
We have added:

- Description of the fidelity checklist used.
- Qualifications and experience of supervising coaches.
- Individualized progression criteria for AEL load (10–20% BM) and drop-jump height based on technique, RPE, and safety.
- Adherence and deviation tracking procedures.
- A sample session log and fidelity checklist provided as Supporting Information.

7. Ethics and Consent Details

Comment:
IEC approval number, CTRI registration details, and consent handling must be clarified.

Response:
We have added:

- IEC approval number/reference.
- CTRI registration ID, registration date, and URL.
- Confirmation that written informed consent will be obtained.
- Storage and retention procedures for consent forms.

8. Editorial and Formatting Issues

Comment:
Inconsistencies in citations, numerals, and figures.

Response:
All editorial issues have been corrected:

- Citation punctuation standardized.
- Numerals formatted consistently per journal style.
- Corrected SPIRIT and CONSORT figures uploaded as high-resolution files.
- All Figshare links verified and functional.

Reviewer #4

We thank Reviewer #4 for the comprehensive and rigorous critique, which has substantially improved the manuscript.

Major Weakness 1: Training Load and Intensity Mismatch

Comment:
Matching ground contacts does not equate mechanical or neuromuscular load.

Response:
We agree with this critique. The manuscript has been revised to:

- Clarify that matching ground contacts was intended to standardize session structure and exposure time, not to imply biomechanical equivalence.
- Explicitly acknowledge in the Discussion (Limitations section) that:
    - Group A receives higher external and internal load.
    - The trial evaluates the added effect of high-intensity eccentric–plyometric loading, not isolated eccentric load.
- Reframe interpretation accordingly, aligning with real-world applied training contexts.

Major Weakness 2: Citation and Reference Quality

Comment:
Use of Google Search URLs and ResearchGate links is unacceptable.

Response:
All such references have been removed and replaced with proper peer-reviewed journal citations with DOIs. The entire reference list has been rechecked and reformatted.

Major Weakness 3: Statistical Reporting

Comment:
Sample size calculation lacks citation and is poorly formatted.

Response:
The sample size section has been corrected, cited, reformatted using standard equation presentation, and all symbols defined.

Minor Weaknesses and Specific Line-by-Line Comments

All points raised have been addressed as follows:

- Abstract phrasing revised to formal academic tone.
- Blinding clarified (assessor-blinded).
- Introduction strengthened with recent biomechanical and eccentric training literature, including all suggested 2025 references.
- Novelty claims regarding AEL verified and revised for accuracy.
- Rationale for combining AEL CMJ and DJ explicitly justified based on complementary SSC adaptations.
- SPIRIT and CONSORT figures corrected and embedded properly.
- Intervention tables clarified regarding ladder volume and additional blocks.
- Agility T-Test limitations acknowledged with citation of advanced multidirectional agility testing.
- Floor/ceiling effect threshold cited.
- Multiple imputation method specified.
- Rehabilitation relevance expanded using modern joint-by-joint training paradigms.
- All problematic references (Refs 18, 21, 29) corrected

Conclusion

We sincerely thank Respected Reviewers #3 and #4 for their detailed and constructive feedback. We believe the revised manuscript now meets the methodological, statistical, and editorial standards required for publication in *PLOS ONE*.

Kind regards, Darpan Chaudhari (on behalf of all co-authors)
Ravi Nair Physiotherapy College
Datta Meghe Institute of Higher Education and Research
Wardha, Maharashtra, India
